# Stress, anxiety, and depression trajectories during the “first wave” of the COVID-19 pandemic: what drives resilient, adaptive and maladaptive responses in the Portuguese population?

**DOI:** 10.3389/fpubh.2024.1333997

**Published:** 2024-02-13

**Authors:** Afonso Fernandes, Sónia Ferreira, Pedro Moreira, Mafalda Machado-Sousa, Beatriz Couto, Catarina Raposo-Lima, Patrício Costa, Pedro Morgado, Maria Picó-Pérez

**Affiliations:** ^1^Life and Health Sciences Research Institute (ICVS), University of Minho, Braga, Portugal; ^2^ICVS/3B's, PT Government Associate Laboratory, Braga, Portugal; ^3^Clinical Academic Center - Braga, Braga, Portugal; ^4^Psychological Neuroscience Lab, CIPsi, School of Psychology, University of Minho, Braga, Portugal

**Keywords:** SARS-CoV-2, psychometrics, mental health, socioeconomic factors, Portugal

## Abstract

**Introduction:**

The COVID-19 outbreak and the community mitigation strategies implemented to reduce new SARS-CoV-2 infections can be regarded as powerful stressors with negative consequences on people's mental health. Although it has been shown that negative emotional symptoms subside during lockdown, it is likely the existence of inter-individual differences in stress, anxiety and depression trajectories throughout lockdown.

**Objectives:**

We aimed to cluster participants' according to their trajectories of stress, anxiety and depression scores throughout lockdown, and identify the sociodemographic, clinical, and lifestyle factors that may distinguish the subjects included in the different clusters.

**Methods:**

From March 23, 2020, to May 31, 2020, participants completed weekly online questionnaires on sociodemographic information (age, sex, education level, and employment status), psychological functioning (DASS-21, NEO-FFI-20), and clinical data (psychiatric disorders, psychiatric medication, physical disorders). Data regarding smoking status, alcohol consumption, physical activity, and time spent daily looking for COVID-19-related information were also collected. Stress, anxiety and depression trajectories were determined using latent class mixed models.

**Results:**

A total of 2040 participants answered the survey at baseline and 603 participants answered all surveys. Three groups (“Resilient,” “Recovered,” and “Maladaptive”) with distinct mental health trajectories were identified. Younger participants, women, participants with lower education level, not working, studying, diagnosed with a mental disorder, taking psychiatric medication, smokers, those who spent more time consuming COVID-19-related information and those with higher neuroticism tended to cluster in the “Maladaptive” group, placing them at higher risk of persistent negative emotional symptoms during compulsory confinement.

**Conclusion:**

Accordingly, a tailored approach to emotional suffering for vulnerable subjects during the COVID-19 and future pandemics must be devised.

## 1 Introduction

The public health crisis caused by COVID-19 forced the implementation of community mitigation strategies to reduce the number of new SARS-CoV-2 infections and prevent the collapse of healthcare systems. Community interventions included social distancing measures, home quarantine, closing of schools and businesses, and travel restrictions (1, 2). In addition, most governments determined periods of compulsory confinement that applied to every citizen, which became known as “lockdowns”: periods that required citizens to stay at home and refrain from or limit social and economic activities outside (3). In Portugal, the first COVID-19 cases were confirmed on the 2nd of March 2020 (4) and on the March 19, 2020 the Portuguese government implemented State of Emergency measures, i.e., social distancing, preventive social isolation or compulsory confinement (5).

While the applied public health measures were effective in reducing new infections and relieving pressure on healthcare systems, they radically changed the lives of those who experienced them. Until now, literature has documented various aspects that seem to affect the psychological wellbeing of the population during lockdown (6, 7). Duration of quarantine, fear of infection, frustration, boredom, and the inability to secure essential goods are well-known stressors (8). Moreover, the socioeconomic impact of the pandemic can be a powerful and long lasting stressor. With people unable to work, rising unemployment, and a drastic decrease in demand in some sectors of the economy, loss of income plays a key role in psychological distress during lockdown (8–10). In fact, increased stress levels and depressive symptoms were found in individuals who reported that COVID-19 influenced their financial situation (11).

Studies on the effect of COVID-19 lockdown on mental health suggest the presence of risk and protective factors associated with stress, anxiety and depression. Younger individuals and women presented increased stress, anxiety and depressive symptoms (2, 11–13). Additionally, lower levels of formal education and previous diagnoses of psychiatric disorder were associated with increased risk of psychiatric symptoms during the COVID-19 pandemic (12, 13). Moreover, an increase in negative emotional symptoms during COVID-19 lockdown was found in individuals who exercised less and who reported poor sleep quality (11). Yet, those diagnosed with arterial hypertension, respiratory diseases or autoimmune disorders showed no changes in negative emotional symptoms during lockdown (11).

However, despite the social, economic and psychological impact of lockdown, it proved to be an effective measure in reducing mortality from COVID-19 (14). Also, the psychological burden of uncontrolled spread of the disease might be worse than that of quarantine (15).

To date, a large number of studies have focused on the impact of the pandemic and lockdown on mental health (3, 16, 17). However, many of these works assume that the emotional response to the pandemic does not vary between subjects. In fact, research shows that there was heterogeneity in mental health response to the COVID-19 pandemic (18). More, pandemics are dynamic events and stress, anxiety and depression scores can vary over time (19, 20). In accordance with the assumption that psychological adaptation to a challenging circumstance is subject to change over time, numerous investigations have explored mental health trajectories during lockdown (19, 21–27).

Repeated measures collected from a sample of the Portuguese population during the first lockdown assessing stress, anxiety and depression using Depression Anxiety and Stress Scale-21 (DASS-21) show us that, in general, negative emotional symptoms diminished throughout lockdown (28). However, as shown in the abovementioned studies, it is presumable that different individuals would show differential trajectories of stress, anxiety and depression scores throughout lockdown. Therefore, we hypothesize that three different trajectory patterns can be found: individuals who sustained low scores of negative emotional symptoms throughout lockdown (“Resilient”); those who presented high scores of negative emotional symptoms at the beginning of lockdown which decreased during the following weeks (“Recovered“); and those who sustained (or increased) high scores of negative emotional symptoms throughout lockdown (“Maladaptive“). Since social isolation and other community mitigation strategies were essential to fight the COVID-19 pandemic, it is of the utmost importance to identify the groups who are more vulnerable to the psychological stressors of lockdown.

By using a latent class mixed model (LCMM) to achieve profile clustering with longitudinal data, this work employs a novel approach to study the impact of COVID-19 lockdown on mental health, focusing on discriminating differences in trajectory shape.

Accordingly, we conducted a longitudinal study to (1) cluster participants according to their time trajectories of stress, anxiety and depression scores throughout lockdown and (2) identify the sociodemographic, clinical, and lifestyle factors that characterize the subjects included in the different clusters. We hope that these analyses help identify those at higher risk for sustained emotional suffering during similar public health crises.

## 2 Materials and methods

### 2.1 Study design

A series of online surveys were used to characterize demographic, social, health and personality variables (15) in a sample of the Portuguese population during enforced social isolation due to the COVID-19 pandemic. The surveys were applied to the general adult Portuguese population from March 23, 2020, to May 31, 2020, starting >1 week after the Portuguese Government announced the first emergency state. The participants completed the questionnaires at baseline (Week 0) and were followed up to 8 times until the lifting of the state of emergency. At each week, a new survey was sent to the participants. DASS-21 scores were collected at nine different time-points along with the date on which the questionnaire response was submitted. The remaining variables were collected only at baseline.

### 2.2 Procedure and measures

Online surveys were applied using Google Forms (Google LLC, USA) and assessed sociodemographic information (age, sex, education level, and employment status), psychological functioning, data regarding housing conditions (access to a terrace and/or garden in the house) and clinical data (presence of psychiatric disorder, psychiatric medication, and having a diagnosis of a physical disorder). Data regarding smoking status, alcohol consumption and practice of physical activity were also collected. In addition, the survey assessed the amount of time spent daily looking for COVID-19-related information.

The psychological assessment of the participants was performed using the Portuguese version of Depression, Anxiety and Stress Scale-21 (DASS-21) and the Portuguese version of NEO-Five Factor Inventory (NEO-FFI-20).

Since stress, anxiety, and depression cannot be measured directly, we used DASS-21, a psychometric test, to assess these latent variables quantitatively (with error). DASS-21 (29, 30) consists of 21 items grouped in 3 subscales that evaluate symptoms related to depression, anxiety and stress experienced in the prior week, and higher scores indicate more negative emotional states. Each item consists of a four-point Likert scale, ranging from 0 (“Did not apply to me at all”) to 3 (“Applied to me very much, or most of the time”). The internal consistency of the Portuguese version of DASS-21 is reflected in the Cronbach's alpha values for depression, anxiety, and stress subscales, which are 0.85, 0.74, and 0.81, respectively. These values suggest a strong internal consistency (30).

NEO-FFI-20 (31, 32) was used to assess differences in personality, with the five subscales of the questionnaire representing the five domains of personality: neuroticism, extraversion, openness to experience, agreeableness, and conscientiousness. Cronbach's alphas for the Portuguese version of the NEO-FFI-20 subscales are consistently high, all above 0.70, indicating a good internal consistency (31).

### 2.3 Participants

The participants were invited through institutional e-mail lists, social media and local and national newspapers. Snowball sampling strategy was used to recruit participants. The eligible population included those 18 years old or older and those capable of understanding the informed consent and questionnaire. Every participant gave informed consent before filling out the questionnaire. Five subjects were excluded for being under 18 years old and four subjects refused to give their consent.

### 2.4 Statistical analyses

Statistical analyses were conducted with R (The R Foundation; version 4.1.0, 2021-05-18), Rstudio (Version 1.4.1717, 2009-21), the IBM SPSS Statistics software (IBM Corp, USA; version 27.0), and Microsoft Excel (Microsoft Excel for Mac, Version 15.30).

The *lcmm* package for R (33) was used to perform an exploratory cluster analysis based on differences in longitudinal trajectories (33). Figures were produced using the *ggplot2* package for R (34). The remaining analyses were conducted in SPSS.

The alpha-value for statistical significance was set to 0.017, corresponding to 0.05 with a Bonferroni correction for three repetitions of all statistical tests for DASS-21 depression, anxiety, and stress scores.

The LCMM method was used to achieve a model-based longitudinal clustering of participant profiles in different groups according to their temporal evolution in DASS-21 depression, anxiety and stress scores (33, 35, 36).

The *time* variable (in days) was computed by subtracting the submission date of the first questionnaire from the submission date of each weekly questionnaire. Additionally, the variable *Time to lockdown* (TTL; in days) was computed by subtracting the submission date of the first questionnaire from the date of March 19, 2020 (the date on which the Portuguese government announced the emergency state). Since participants did not all submit the baseline survey on the same day, the TTL variable was computed to minimize differences arising from variation at the beginning of follow up.

The change over time of the latent process underlying the DASS-21 subscale scores was described using a two-sided formula. The fixed-effects in the linear mixed model were defined using *time* and *time*^2^ (quadratic term) and using *TTL* as a covariate. The variable *time* was defined as a class-specific regression parameter and as having a random effect. The random effects were grouped by participant, to account for variability among participants in the sample due to causes that are not being equated in the model. This was applied to all LCMM models regarding the stress, depression and anxiety DASS-21 subscale scores.

From our main hypothesis, based on recent literature (19, 21, 37), we expected three different trajectories in the evolution of stress, anxiety and depression scores throughout lockdown: “Resilient,” “Recovered,” and “Maladaptive” (21). Therefore, the number of clusters/classes was defined as 3. Other numbers of clusters (2 and 4) were also explored. Labels were selected according to the ones used in analogous work on mental health trajectories during adversity (19, 21, 37). Models for the same number of classes with different link functions were estimated. Every model was estimated using an unstructured and a diagonal matrix of variance-covariance. The latent process model with three-class solution with the lowest discrete Akaike information criterion (discrete AIC) value was considered the best fit (38). Participants were clustered based on the participant's most likely latent class membership.

To identify which sociodemographic, clinical, and lifestyle factors characterize the subjects included in the different clusters, the normality of continuous variables was first assessed using Shapiro-Wilk's test. When the tested variable was not normally distributed, the Kruskal–Wallis *H*-test was used to assess age differences, levels of formal education and NEO-FFI subscale scores among the three different clusters of participants identified with the LCMM model. When the Kruskal–Wallis *H*-test indicated a significance level, the *post-hoc* Dunn's multiple comparison test was used to compare all pairs of clusters. The significance values of Dunn's multiple comparison test were adjusted using the Bonferroni correction. Here, we multiplied the *p-*value by three, the total number of pairwise comparisons (Maladaptive vs. Recovered, Maladaptive vs. Resilient, Recovered vs. Resilient).

Pearson's chi-square test was used to assess differences among participants in the three clusters involving the following categorical variables: sex, employment status, having a diagnosis of a psychiatric disorder, taking psychiatric medication, having a diagnosis of a physical disorder, smoking, alcohol consumption, having access to a terrace and/or garden in the house, practicing physical activity/exercise, amount of time spent daily exposed to COVID-19 related news. When the Pearson's chi-square test was deemed significant, the adjusted residual values of each cell were used to derive the *p*-value using the CHISQ.DIST.RT function in Microsoft Excel, taking into account the number of multiple comparisons performed (39).

### 2.5 Ethical statement

The ethical committee approved this study from the Ethics Committee for Research in Life and Health Sciences (CEIVCS). Electronic informed consent was obtained from all the participants. The study aims were comprehensively explained, and the participants could withdraw from the study at any moment without being harmed in the relationship with the team of researchers. Apart from the time required to answer the questionnaires, this study did not have any cost or risks for the participants.

A unique code was generated for each participant to maintain their anonymity and the confidentiality of their answers. All the information was collected and treated in a confidential, anonymized and coded manner.

This study was carried out following the Helsinki Declaration (2008), the European Convention on Human Rights and Biomedicine (1997), the Council for International Medical Science Organizations (1993), and the Guide to Good Clinical Practice (2000).

## 3 Results

### 3.1 Demographic characterization of the population in the sample

A total of 2,040 participants answered the survey at baseline (Week 0). Due to dropout, 1,446 (70.9%) of these participants answered to the survey at Week 1, 1,302 (63.8%) at Week 2, 1,266 (62.1%) at Week 3, 1,183 (58.0%) at Week 4, 1111 (54.5%) at Week 5, 1,046 (51.3%) at Week 6, 1,058 (51.9%) at Week 7, and 1,020 (50.0%) at Week 8. 603 (29, 6%) participants answered all surveys. The total sample (*n* = 2,040 participants), among whom 1,650 (80.9%) were women and 390 (19.1%) were men, had a mean age of 38.04 [standard deviation (SD) 12.19] with a range between 18 and 88 years old. The mean number of completed years of education was 15.69 (SD 2.55). Although the participants were largely well-educated, this sample's years of formal education ranged from 4 (primary school) to 21 (doctorate). In addition, 1, 309 (64.2%) of the participants were working, 246 (12.1%) were studying and 485 (23.8%) were unemployed. Table 1 describes all the study variables for the total sample.

**Table 1:** Descriptive statistics of the variables for the 2, 040 participants at baseline assessment.

***n***	**2, 040**
Age, mean (SD) | median (IQR)	38.04 (12.19) | 37 (18)
Level of education, mean (SD) | median (IQR)	15.69 (2.55) | 15 (2)
**Sex**, ***n*** **(%)**	
Female	1, 650 (80.9%)
Male	390 (19.1%)
**Employment status**, ***n*** **(%)**	
Unemployed	485 (23.8%)
Studying	246 (12.1%)
Working	1, 309 (64.2%)
**Balcony/Terrace**, ***n*** **(%)**	
No	252 (12.4%)
Yes	1, 788 (87.6%)
**Psychiatric disorder**, ***n*** **(%)**	
No	1, 787 (87.6%)
Yes	253 (12.4%)
**Psychiatric medication**, ***n*** **(%)**	
No	1, 652 (81.0%)
Yes	388 (19.0%)
**Physical disorder**, ***n*** **(%)**	
No	1, 345 (65.9%)
Yes	695 (34.1%)
**Smoking**, ***n*** **(%)**	
No	1, 608 (78.8%)
Yes	432 (21.2%)
**Alcohol consumption**, ***n*** **(%)**	
No	1, 748 (85.7%)
Yes	292 (14.3%)
**Physical activity**, ***n*** **(%)**	
No	744 (36.5%)
Yes	1, 296 (63.5%)
**COVID-19 Time**, ***n*** **(%)**	
< 1 h	1, 192 (58.4%)
1 h or more	848 (41.6%)
**NEO-FFI, mean (SD) | median (IQR)**	
Neuroticism	7.79 (3.33) | 8 (5)
Extraversion	8.86 (2.80) | 9 (4)
Openness to experience	10.42 (3.61) | 11 (5)
Agreeableness	10.46 (3.07) | 11 (4)
Conscientiousness	11.61 (2.58) | 12 (3)

n, number of participants; SD, standard deviation; IQR, interquartile range.

### 3.2 Longitudinal clustering of the sample according to DASS-21 scores

**Figure 1:**
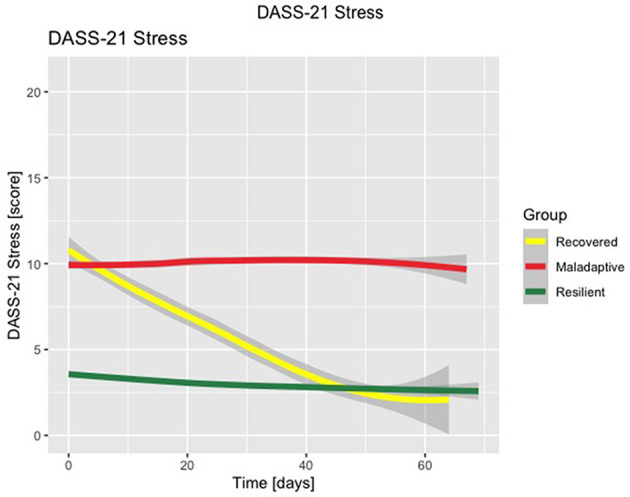
Latent class mixed model with three-class solution for DASS-21 Stress subscale scores. Time is displayed in number of days.

**Figure 2:**
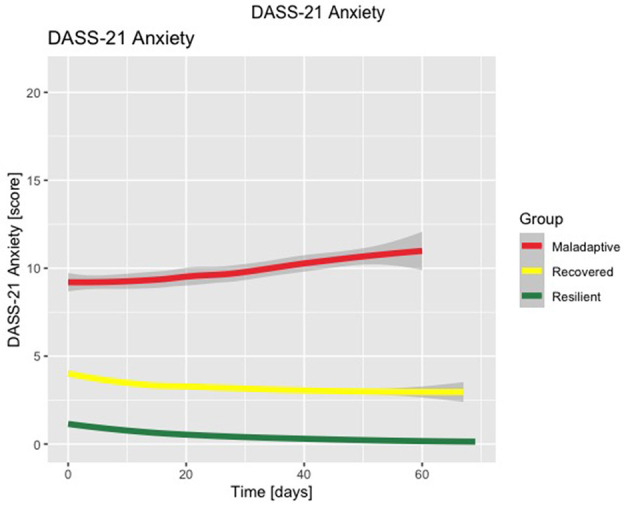
Latent class mixed model with three-class solution for DASS-21 Anxiety subscale scores. Time is displayed in number of days.

**Figure 3:**
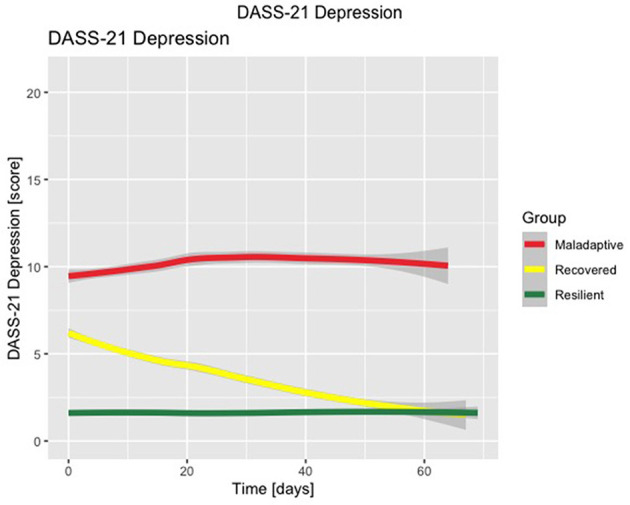
Latent class mixed model with three-class solution for DASS-21 Depression subscale scores. Time is displayed in number of days.

The three-class solution LCMM with the best fit for the DASS-21 Stress subscale (discrete AIC = 51683.78, number of parameters = 18), DASS-21 Anxiety subscale (discrete AIC = 35598.85, number of parameters = 17), and DASS-21 Depression subscale (discrete AIC = 43569.12, number of parameters = 18) are shown in Figures 1–3, respectively. Supplementary Tables 1–3 detail fit indices for all estimated models. No substantial improvements in fit indices were found in models with two and four-class solutions. The participants were clustered in 3 different groups based on the participant's most likely latent class membership, according to the temporal trajectory of DASS-21 subscales scores during lockdown. Regarding the DASS-21 Stress subscale, despite one of the clusters having < 5% of the sample, the two-cluster solution did not provide a better fit.

### 3.3 Comparison of baseline characteristics among clusters

The continuous variables age and the five NEO-FFI subscales (neuroticism, extraversion, openness, agreeableness, and conscientiousness) were not normally distributed. When the Shapiro-Wilk test was applied, the null hypothesis was rejected for all the previously mentioned variables. Similarly, the variable reflecting the level of formal education (an ordinal variable) presented a non-normal distribution. Therefore, the Kruskal–Wallis *H-*test assessed differences in the mentioned variables between the three different clusters.

### 3.4 Differences among DASS-21 stress clusters

The latent process model with the best fit for the DASS-21 Stress subscale provided three different clusters: the Maladaptive cluster with 854 (42%) subjects, the Recovered cluster with 74 (4%) subjects, and the Resilient cluster with 1,112 (55%) subjects. After obtaining the clusters of participants based on the trajectories of DASS-21 stress scores throughout lockdown, these clusters were compared to each other to identify possible risk and protective factors for mental wellbeing during lockdown. Table 2 summarizes the differences among DASS-21 Stress clusters.

**Table 2:** Cluster demographics and comparison of the three latent classes for DASS-21 stress subscale.

**Variable**	**Maladaptive**	**Recovered**	**Resilient**	**Statistical test**	***Post-hoc* tests**
*N*	854 (41.86%)	74 (3.63%)	1, 112 (54.51%)	-	-
Age, mean (SD) | median (IQR)	36.06 (11.19) | 35 (16)	35.72 (10.24) | 34.5 (15)	39.71 (12.79) | 38 (19)	H (2) = 40.147 *p* < 0.001^*^	Maladaptive vs. Recovered: *p* > 0.999^**^ | Recovered vs. Resilient: *p* = 0.041^**^ | Maladaptive vs. Resilient: *p* < 0.001^*^|^**^
Level of education (SD) | median (IQR)	15.57 (2.53) | 15 (2)	15.99 (2.37) | 15 (2)	15.76 (2.57) | 15 (2)	H(2) = 4.108 *p* = 0.128	-
**Sex**, ***N*** **(%)**					
Female	724 (84.8%)	70 (94.6%)	856 (77.0%)	*X^2^*(2) = 28.338 *p* < 0.001^*^	Maladaptive: *p* < 0.001^*^ | Recovered: *p* = 0.012^*^ | Resilient: *p* < 0.001^*^
Male	130 (15.2%)	4 (5.4%)	256 (23.0%)		
**Employment status**, ***N*** **(%)**					
Unemployed	220 (25.8%)	15 (20.3%)	250 (22.5%)	*X^2^*(4) = 12.456 *p* = 0.014^*^	Unemployed: Maladaptive: *p* = 0.647 | Recovered: *p* > 0.999 | Resilient: *p* = 1.000
Studying	120 (14.1%)	5 (6.8%)	121 (10.9%)		Studying: Maladaptive: *p* = 0.193 | Recovered: *p* > 0.999 | Resilient: *p* = 0.647
Working	514 (60.2%)	54 (73.0%)	741 (66.6%)		Working: Maladaptive: *p* = 0.012^*^ | Recovered: *p* = 0.986 | Resilient: *p* = 0.112
**Balcony/Terrace**, ***N*** **(%)**					
No	123 (14.4%)	15 (20.3%)	114 (10.3%)	*X^2^*(2) = 12.133 *p* = 0.002^*^	Maladaptive: *p* = 0.098 | Recovered: *p* = 0.214 | Resilient: *p* = 0.008^*^
Yes	731 (85.6%)	59 (79.7%)	998 (89.7%)		
**Psychiatric disorder**, ***N*** **(%)**					
No	693 (81.1%)	63 (85.1%)	1, 031 (92.7%)	*X^2^*(2) = 59.931 *p* < 0.001^*^	Maladaptive: *p* < 0.001^*^ | Recovered: *p* > 0.999 | Resilient: *p* < 0.001^*^
Yes	161 (18.9%)	11 (14.9%)	81 (7.3%)		
**Psychiatric medication**, ***N*** **(%)**					
No	634 (74.2%)	59 (79.7%)	959 (86.2%)	*X^2^*(2) = 45.255 *p* < 0.001^*^	Maladaptive: *p* < 0.001^*^ | Recovered: *p* > 0.999 | Resilient: *p* < 0.001^*^
Yes	220 (25.8%)	15 (20.3%)	153 (13.8%)		
**Physical disorder**, ***N*** **(%)**					
No	558 (65.3%)	54 (73.0%)	733 (65.9%)	*X^2^*(2) = 1.767 *p* = 0.413	-
Yes	296 (34.7%)	20 (27.0%)	379 (34.1%)		
**Smoking**, ***N*** **(%)**					
No	640 (74.9%)	56 (75.7%)	912 (82.0%)	*X^2^*(2) = 14.933 *p* < 0.001^*^	Maladaptive: *p* = 0.002^*^ | Recovered: *p* > 0.999 | Resilient: *p* < 0.001^*^
Yes	214 (25.1%)	18 (24.3%)	200 (18.0%)		
**Alcohol consumption**, ***N*** **(%)**					
No	739 (86.5%)	62 (83.8%)	947 (85.2%)	*X^2^*(2) = 0.968 *p* = 0.616300	-
Yes	115 (13.5%)	12 (16.2%)	165 (14.8%)		
**Physical activity**, ***N*** **(%)**					
No	364 (42.6%)	33 (44.6%)	347 (31.2%)	*X^2^*(2) = 29.366 *p* < 0.001^*^	Maladaptive: *p* < 0.001^*^ | Recovered: *p* = 0.802 | Resilient: *p* < 0.001^*^
Yes	490 (57.4%)	41 (55.4%)	765 (68.8%)		
**COVID-19 time**, ***N*** **(%)**					
< 1 h	457 (53.5%)	31 (41.9%)	704 (63.3%)	*X^2^*(2) = 27.734 *p* < 0.001^*^	Maladaptive: *p* < 0.001^*^ | Recovered: *p* = 0.022 | Resilient: *p* < 0.001^*^
More than 1 h	397 (46.5%)	43 (58.1%)	408 (36.7%)		
NEO-FFI neuroticism, mean (SD) | median (IQR)	9.82 (2.70) | 10 (4)	8.49 (2.97) | 9 (4)	6.28 (2.97) | 6 (4)	H(2) = 351.412 *p* < 0.001^*^	Maladaptive vs. Recovered: *p* = 0.007^*^|^**^ | Recovered vs. Resilient: *p* < 0.001^*^|^**^ | Maladaptive vs. Resilient: *p* < 0.001^*^|^**^
NEO-FFI extraversion, mean (SD) | median (IQR)	8.00 (2.83) | 8 (4)	9.18 (3.24) | 9 (4)	9.45 (2.58) | 10 (3)	H(2) = 82.716 *p* < 0.001^*^	Maladaptive vs. Recovered: *p* = 0.005^*^|^**^ | Recovered vs. Resilient: *p* > 0.999^**^ | Maladaptive vs. Resilient: *p* < 0.001^*^|^**^
NEO-FFI openness to experience, mean (SD) | median (IQR)	10.23 (3.79) | 11 (5)	10.44 (3.76) | 10 (5)	10.56 (3.46) | 11 (5)	H(2) = 1.066 *p* = 0.587	-
NEO-FFI agreeableness, mean (SD) | median (IQR)	9.93 (3.05) | 10 (4)	10.40 (3.22) | 11 (5)	10.84 (3.02) | 11 (4)	H(2) = 28.877 *p* < 0.001^*^	Maladaptive vs. Recovered: *p* = 0.407^**^ | Recovered vs. Resilient: *p* > 0.999^**^ | Maladaptive vs. Resilient: *p* < 0.001^*^|^**^
NEO-FFI conscientiousness, mean (SD) | median (IQR)	11.18 (2.63) | 12 (3)	11.15 (3.04) | 12 (3)	11.96 (2.46) | 12 (2)	H(2) =23.570 *p* < 0.001^*^	Maladaptive vs. Recovered: *p* > 0.999^**^ | Recovered vs. Resilient: *p* = 0.423^**^ | Maladaptive vs. Resilient: *p* < 0.001^*^|^**^

P-values for post hoc tests were corrected with Bonferroni correction using the number of comparisons performed. ^*^Statistically significant result for p < 0.017. ^**^The significance values of Dunn's multiple comparison test were adjusted using the Bonferroni correction.

This analysis revealed that the Maladaptive and Resilient clusters differed in age, with the Maladaptive cluster having younger participants than the Resilient cluster. Additionally, our results show that these clusters differed in sex, with a higher percentage of men in the Resilient cluster, followed by the Maladaptive cluster.

We found that the Maladaptive cluster had more participants diagnosed with a psychiatric disorder and taking psychiatric medication.

Furthermore, our research shows that the Resilient cluster had more non-smokers, more subjects who exercise regularly, and fewer individuals spending more than 1 h per day exposed to COVID-19 related news.

Finally, the Maladaptive cluster had fewer employed participants. In contrast, the Resilient cluster differed in the access to a green space or a balcony in the house, with more individuals living in houses with access to a balcony or terrace.

Personality differences were also compared between clusters using NEO-FFI-20 and its five subscales. All clusters differed significantly in the NEO-FFI Neuroticism subscale. Regarding the NEO-FFI Extraversion subscale, the Maladaptive cluster differed significantly from the Recovered and Resilient clusters. The Maladaptive cluster had lower extraversion scores than the Recovered and Resilient clusters. The Maladaptive and Resilient clusters differed in the NEO-FFI Agreeableness and NEO-FFI Conscientiousness subscales, with the Maladaptive cluster presenting lower agreeableness and conscientiousness scores.

### 3.5 Differences among DASS-21 anxiety clusters

The clusters obtained with the best-fitting latent process model for the DASS-21 Anxiety subscale were the following: the Maladaptive cluster with 191 (9%) subjects, the Recovered cluster with 721 (35%) subjects, and the Resilient cluster with 1,128 (55%) subjects. The results on the differences among DASS-21 Anxiety clusters are presented in Table 3.

**Table 3:** Cluster demographics and comparison of the three latent classes for DASS-21 Anxiety subscale.

**Variable**	**Maladaptive**	**Recovered**	**Resilient**	**Statistical test**	***Post-hoc* tests**
*n*	191 (9.36%)	721 (35.34%)	1,128 (55.29%)	-	-
Age, mean (SD) | median (IQR)	34.43 (11.93) | 33 (19)	37.91 (12.36) | 36 (18)	38.73 (12.02) | 38 (17)	H(2) = 24.009 *p* < 0.001^*^	Maladaptive vs. Recovered: *p* < 0.001^*^|^**^ | Recovered vs. Resilient: *p* = 0.209^**^ | Maladaptive vs. Resilient: *p* < 0.001^*^|^**^
Level of education (SD) | Median (IQR)	14.87 (2.38) | 15 (5)	15.53 (2.47) | 15 (2)	15.93 (2.59) | 15 (2)	H(2) = 33.975 *p* < 0.001^*^	Maladaptive vs. Recovered: *p* = 0.014^*^|^**^ | Recovered vs. Resilient: *p* < 0.001^*^|^**^ | Maladaptive vs. Resilient: *p* < 0.001^*^|^**^
**Sex**, ***n*** **(%)**					
Female	166 (86.9%)	598 (82.9%)	886 (78.5%)	*X^2^*(2) = 10.445868 *p* = 0.005391^*^	Maladaptive: *p* = 0.167 | Recovered: *p* = 0.535 | Resilient: *p* = 0.016^*^
Male	25 (13.1%)	123 (17.1%)	242 (21.5%)		
**Employment status**, ***n*** **(%)**					
Unemployed Studying Working	60 (31.4%) 43 (22.5%) 88 (46.1%)	185 (25.7%) 92 (12.8%) 444 (61.6%)	240 (21.3%) 111 (9.8%) 777 (68.9%)	*X^2^*(4) = 45.340 *p* < 0.001^*^	Unemployed: Maladaptive: *p* = 0.084 | Recovered: *p* > 0.999 | Resilient: *p* = 0.034 Studying: Maladaptive: *p* < 0.001^*^ | Recovered: *p* > 0.999 | Resilient: *p* = 0.006^*^ Working: Maladaptive: *p* < 0.001^*^ | Recovered: *p* = 0.647 | Resilient: *p* < 0.001^*^
**Balcony/Terrace**, ***n*** **(%)**					
No	26 (13.6%)	112 (15.5%)	114 (10.1%)	*X^2^*(2) = 12.277 *p* = 0.002^*^	Maladaptive: *p* > 0.999 | Recovered: *p* = 0.008^*^ | Resilient: *p* = 0.004^*^
Yes	165 (86.4%)	609 (84.5%)	1, 014 (89.9%)		
**Psychiatric disorder**, ***n*** **(%)**					
No	126 (66.0%)	615 (85.3%)	1, 046 (92.7%)	*X^2^*(2) = 113.112 *p* < 0.001^*^	Maladaptive: *p* < 0.001^*^ | Recovered: *p* = 0.129 | Resilient: *p* < 0.001^*^
Yes	65 (34.0%)	106 (14.7%)	82 (7.3%)		
**Psychiatric medication**, ***n*** **(%)**					
No	110 (57.6%)	554 (76.8%)	988 (87.6%)	*X^2^*(2) = 107.852 *p* < 0.001^*^	Maladaptive: *p* < 0.001^*^ | Recovered: *p* = 0.003^*^ | Resilient: *p* < 0.001^*^
Yes	81 (42.4%)	167 (23.2%)	140 (12.4%)		
**Physical disorder**, ***n*** **(%)**					
No	114 (59.7%)	451 (62.6%)	780 (69.1%)	*X^2^*(2) = 12.181 *p* = 0.002264^*^	Maladaptive: *p* = 0.345 | Recovered: *p* = 0.098 | Resilient: *p* = 0.004^*^
Yes	77 (40.3%)	270 (37.4%)	348 (30.9%)		
**Smoking**, ***n*** **(%)**					
No	137 (71.7%)	545 (75.6%)	926 (82.1%)	*X^2^*(2) = 17.582 *p* < 0.001^*^	Maladaptive: *p* = 0.075 | Recovered: *p* = 0.042 | Resilient: *p* < 0.001^*^
Yes	54 (28.3%)	176 (24.4%)	202 (17.9%)		
**Alcohol consumption**, ***n*** **(%)**					
No	169 (88.5%)	617 (85.6%)	962 (85.3%)	*X^2^*(2) = 1.373 *p* = 0.503292	-
Yes	22 (11.5%)	104 (14.4%)	166 (14.7%)		
**Physical Activity**, ***n*** **(%)**					
No	93 (48.7%)	307 (42.6%)	344 (30.5%)	*X^2^*(2) = 41.301 *p* < 0.001^*^	Maladaptive: *p* = 0.001^*^ | Recovered: *p* < 0.001^*^ | Resilient: *p* < 0.001^*^
Yes	98 (51.3%)	414 (57.4%)	784 (69.5%)		
**COVID-19 time**, ***n*** **(%)**					
< 1 h	97 (50.8%)	387 (53.7%)	708 (62.8%)	*X^2^*(2) = 20.037 *p* < 0.001^*^	Maladaptive: *p* = 0.129 | Recovered: *p* = 0.008^*^ | Resilient: *p* < 0.001^*^
More than 1 h	94 (49.2%)	334 (46.3%)	420 (37.2%)		
NEO-FFI neuroticism, mean (SD) | median (IQR)	10.86 (2.54) | 11 (4)	9.17 (2.88) | 9 (4)	6.52 (3.07) | 6 (5)	H(2) = 273.498 *p* < 0.001^*^	Maladaptive vs. Recovered: *p* < 0.001^*^|^**^ | Recovered vs. Resilient: *p* < 0.001^*^|^**^ | Maladaptive vs. Resilient: *p* < 0.001^*^|^**^
NEO-FFI extraversion, mean (SD) | median (IQR)	7.17 (2.92) | 7.5 (4)	8.26 (2.78) | 8 (3)	9.47 (2.62) | 10 (3)	H(2) = 91.248 *p* < 0.001^*^	Maladaptive vs. Recovered: *p* = 0.002^*^|^**^ | Recovered vs. Resilient: *p* < 0.001^*^|^**^ | Maladaptive vs. Resilient: *p* < 0.001^*^|^**^
NEO-FFI openness to experience, mean (SD) | median (IQR)	9.64 (4.19) | 10 (7)	10.30 (3.65) | 11 (5)	10.61 (3.48) | 11 (5)	H(2) = 4.147 *p* = 0.125746	-
NEO-FFI agreeableness, mean (SD) | median (IQR)	9.32 (3.56) | 10 (5)	10.03 (2.92) | 10 (4)	10.88 (3.01) | 11 (4)	H(2) = 36.632 *p* < 0.001^*^	Maladaptive vs. Recovered: *p* = 0.275^**^ | Recovered vs. Resilient: *p* < 0.001^*^|^**^ | Maladaptive vs. Resilient: *p* < 0.001^*^|^**^
NEO-FFI conscientiousness, mean (SD) | Median (IQR)	11.07 (3.04) | 12 (3)	11.06 (2.67) | 12 (3)	12.03 (2.38) | 12 (2)	H(2) = 39.784 *p* < 0.001^*^	Maladaptive vs. Recovered: *p* > 0.999^**^ | Recovered vs. Resilient: *p* < 0.001^*^|^**^ | Maladaptive vs. Resilient: *p* = 0.008^*^|^**^

P-values for post hoc tests were corrected with Bonferroni correction using the number of comparisons performed. ^*^Statistically significant result for p < 0.017. ^**^The significance values of Dunn's multiple comparison test were adjusted using the Bonferroni correction.

The analyses performed to assess differences between clusters revealed that the Maladaptive cluster differed significantly in age from the Recovered and Resilient clusters. The Maladaptive cluster had younger participants than the Recovered and Resilient clusters. In addition, the Resilient cluster differed in terms of sex composition, having more males and fewer females.

Moreover, all clusters differed significantly from each other in level of education, with the Maladaptive cluster presenting a lower education level than the Recovered cluster.

Participants in the Maladaptive and Resilient clusters differed in terms of their employment status, with the Maladaptive cluster presented more students and less employed participants.

When we compared the participants' mental wellbeing data, the results showed that the Maladaptive cluster presented more participants diagnosed with a psychiatric disorder. Additionally, all clusters differed in terms of participants taking psychiatric medication.

We found that the Resilient cluster had more participants with access to a balcony or terrace and fewer subjects spending more than 1 h per day exposed to COVID-19 related news. Furthermore, the Resilient cluster displayed significant differences in the number of smokers and participants diagnosed with a physical disorder, having more non-smokers and fewer subjects diagnosed with a physical disorder.

Finally, regarding the practice of physical activity, all clusters differed from each other. The Resilient cluster presented the higher percentage of participants who practice exercise, followed by the Recovered cluster, and the Maladaptive cluster showed the lowest percentage.

Regarding differences in the big five domains of personality, all clusters differed significantly in the NEO-FFI Neuroticism and Extraversion subscales. The Maladaptive and Recovered clusters differed from the Resilient cluster in the NEO-FFI Agreeableness and NEO-FFI Conscientiousness subscales. The Maladaptive and Recovered cluster presented lower agreeableness conscientiousness than the Resilient cluster.

### 3.6 Differences among DASS-21 depression clusters

The best-fitting latent process model for the DASS-21 Depression subscale provided the following three clusters: the Maladaptive cluster with 467 (23%) subjects, the Recovered cluster with 302 (15%) subjects, and the Resilient cluster with 1, 271 (62%) subjects. Table 4 highlights the differences among DASS-21 Depression clusters.

**Table 4:** Cluster demographics and comparison of the three latent classes for DASS-21 Depression subscale.

**Variable**	**Maladaptive**	**Recovered**	**Resilient**	**Statistical test**	***Post-hoc* tests**
*n*	467 (22.89%)	302 (14.80%)	1, 271 (62.30%)	-	-
Age, mean (SD) | median (IQR)	35.29 (12.06) | 33 (19)	36.66 (11.58) | 34.5 (16)	39.38 (12.18) | 38 (17)	H(2) = 48.634 *p* < 0.001^*^	Maladaptive vs. Recovered: *p* = 0.282^**^ | Recovered vs. Resilient: *p* < 0.001^*^|^**^ | Maladaptive vs. Resilient: *p* < 0.001^*^|^**^
Level of education (SD) | Median (IQR)	15.40 (2.53) | 15 (2)	15.61 (2.53) | 15 (2)	15.82 (2.55) | 15 (2)	H(2) = 11.648 *p* = 0.003^*^	Maladaptive vs. Recovered: *p* = 0.721^**^ | Recovered vs. Resilient: *p* = 0.429^**^ | Maladaptive vs. Resilient: *p* = 0.003^*^|^**^
**Sex**, ***n*** **(%)**					
Female	370 (79.2%)	257 (85.1%)	1, 023 (80.5%)	*X^2^*(2) = 4.427	-
Male	97 (20.8%)	45 (14.9%)	248 (19.5%)	*p* = 0.109	
**Employment Status**, ***n*** **(%)**					
Unemployed	139 (29.8%)	78 (25.8%)	268 (21.1%)	*X^2^*(4) = 38.898	Unemployed: Maladaptive: *p* = 0.004^*^ | Recovered: *p* > 0.999 | Resilient: *p* = 0.002^*^
Studying	81 (17.3%)	34 (11.3%)	131 (10.3%)	*p* < 0.001^*^	Studying: Maladaptive: *p* < 0.001^*^ | Recovered: *p* > 0.999 | Resilient: *p* = 0.002^*^
Working	247 (52.9%)	190 (62.9%)	872 (68.6%)		Working: Maladaptive: *p* < 0.001^*^ | Recovered: *p* > 0.999 | Resilient: *p* = 0.002^*^
**Balcony/Terrace**, ***n*** **(%)**					
No	82 (17.6%)	44 (14.6%)	126 (9.9%)	*X^2^*(2) = 20.046 *p* < 0.001^*^	Maladaptive: *p* < 0.001^*^ | Recovered: *p* > 0.999 | Resilient: *p* < 0.001^*^
Yes	385 (82.4%)	258 (85.4%)	1, 145 (90.1%)		
**Psychiatric disorder**, ***n*** **(%)**					
No	353 (75.4%)	268 (88.7%)	1, 167 (91.8%)	*X^2^*(2) = 85.418 *p* < 0.001^*^	Maladaptive: *p* < 0.001^*^ | Recovered: *p* > 0.999 | Resilient: *p* < 0.001^*^
Yes	115 (24.6%)	34 (1.3%)	104 (8.2%)		
**Psychiatric medication**, ***n*** **(%)**					
No	324 (69.4%)	245 (81.1%)	1, 083 (85.2%)	*X^2^*(2) = 55.565 *p* < 0.001^*^	Maladaptive: *p* < 0.001^*^ | Recovered: *p* > 0.999 | Resilient: *p* < 0.001^*^
Yes	143 (30.6%)	57 (18.9%)	188 (14.8%)		
**Physical disorder**, ***n*** **(%)**					
No	290 (62.1%)	206 (68.2%)	849 (66.8%)	*X^2^*(2) = 4.178 *p* = 0.123787	-
Yes	177 (37.9%)	96 (31.8%)	422 (33.2%)		
**Smoking**, ***n*** **(%)**					
No	344 (73.7%)	230 (76.2%)	1, 034 (81.4%)	*X^2^*(2) = 13.612 *p* = 0.001107^*^	Maladaptive: *p* = 0.012^*^ | Recovered: *p* > 0.999 | Resilient: *p* = 0.002^*^
Yes	123 (26.3%)	72 (23.8%)	237 (18.6%)		
**Alcohol consumption**, ***n*** **(%)**					
No	401 (85.9%)	257 (85.1%)	1, 090 (85.8%)	*X^2^*(2) = 0.103 *p* = 0.949893	-
Yes	66 (14.1%)	45 (14.9%)	181 (14.2%)		
**Physical activity**, ***n*** **(%)**					
No	212 (45.4%)	117 (38.7%)	415 (32.7%)	*X^2^*(2) = 24.731 *p* < 0.001^*^	Maladaptive: *p* = 0.002^*^ | Recovered: *p* > 0.999 | Resilient: *p* < 0.001^*^
Yes	255 (54.6%)	185 (61.3%)	856 (67.3%)		
**COVID-19 time**, ***n*** **(%)**					
>1 h	243 (52.0%)	158 (52.3%)	791 (62.2%)	*X^2^*(2) = 20.084 *p* < 0.001^*^	Maladaptive: *p* = 0.008^*^ | Recovered: *p* > 0.999 | Resilient: *p* < 0.001^*^
More than 1 h	224 (48.0%)	144 (47.7%)	480 (37.8%)		
NEO-FFI Neuroticism, mean (SD) | median (IQR)	11.16 (2.39) | 11 (4)	8.73 (2.71) | 9 (4)	6.44 (2.83) | 6 (4)	H(2) = 431.912 *p* < 0.001^*^	Maladaptive vs. Recovered: *p* < 0.001^*^|^**^ | Recovered vs. Resilient: *p* < 0.001^*^|^**^ | Maladaptive vs. Resilient: *p* < 0.001^*^|^**^
NEO-FFI extraversion, mean (SD) | median (IQR)	6.91 (2.98) | 7 (4)	8.90 (2.79) | 9 (4)	9.50 (2.42) | 10 (3)	H(2) = 160.775 *p* < 0.001^*^	Maladaptive vs. Recovered: *p* < 0.001^*^|^**^ | Recovered vs. Resilient: *p* = 0.006^*^|^**^ | Maladaptive vs. Resilient: *p* < 0.001^*^|^**^
NEO-FFI openness to experience, mean (SD) | median (IQR)	9.90 (3.75) | 10 (6)	10.35 (4.20) | 10.5 (5)	10.62 (3.38) | 11 (5)	H(2) = 7.113 *p* = 0.028544	-
NEO-FFI agreeableness, mean (SD) | median (IQR)	9.54 (3.13) | 10 (4)	10.48 (3.39) | 11 (5)	10.77 (2.91) | 11 (4)	H(2) = 34.991 *p* < 0.001^*^	Maladaptive vs. Recovered: *p* = 0.005^*^|^**^ | Recovered vs. Resilient: *p* = 0.391 | Maladaptive vs. Resilient: *p* < 0.001^*^|^**^
NEO-FFI conscientiousness, mean (SD) | median (IQR)	10.37 (3.02) | 11 (3)	11.41 (2.75) | 12 (3)	12.08 (2.22) | 12 (2)	H(2) = 74.893 *p* < 0.001^*^	Maladaptive vs. Recovered: *p* < 0.001^*^|^**^ | Recovered vs. Resilient: *p* = 0.004^*^|^**^ | Maladaptive vs. Resilient: *p* < 0.001^*^|^**^

P-values for post-hoc tests were corrected with Bonferroni correction using the number of comparisons performed. ^*^Statistically significant result for p < 0.017. ^**^The significance values of Dunn's multiple comparison test were adjusted using the Bonferroni correction.

The analysis performed here revealed that the Maladaptive and Recovered clusters had younger participants than the Resilient cluster. Furthermore, the Maladaptive cluster presented a lower number of completed years of education than the Resilient cluster.

Moreover, the differences in employment status between the Maladaptive and Resilient clusters were evident. The Maladaptive cluster presented more unemployed participants, more students and fewer employed participants.

The Maladaptive cluster had more participants diagnosed with a psychiatric disorder and taking psychiatric medication.

In addition, the analyses revealed that the Maladaptive cluster had more smokers and fewer participants who practice physical activity.

Furthermore, we found that the Resilient cluster had more participants with access to a balcony or terrace and fewer subjects spending more than 1 h per day exposed to COVID-19 related news.

All clusters differed significantly in the NEO-FFI neuroticism, extraversion, and consciousness subscales. Finally, the Maladaptive cluster differed from the Recovered and Resilient cluster in the NEO-FFI Agreeableness subscale, with the Maladaptive cluster presenting lower agreeableness than the Recovered and Resilient clusters.

## 4 Discussion

The longitudinal studies analyzing stress, anxiety and depression symptoms during COVID-19 compulsory confinement, including one study carried out in the Portuguese population, are unanimous in recognizing that the stress, anxiety and depression decreased linearly over lockdown (28, 40–42).

However, individuals differ in countless aspects, and many of these differences can influence adaptation to changing demands. Therefore, due to the existing heterogeneity among individuals, and according to the available evidence (19, 21–27), it is presumable that the linear decrease in DASS-21 subscale scores presents an oversimplification of individual trajectories, and that different individuals display different trajectories in the evolution of stress, anxiety and depression scores throughout lockdown.

Hence, our work aimed to explore trajectories of stress, anxiety, and depression symptoms across the first lockdown response to the COVID-19 pandemic in Portugal. Additionally, this study intended to determine the factors that characterize individuals with different trajectories and thus explain the differences in the evolution of stress, anxiety and depression scores throughout lockdown.

Taken together, the findings of this work suggest that participants can be clustered in three groups with distinct mental health trajectories: a “Resilient” group with sustained low scores of negative emotional symptoms throughout lockdown, a “Recovered” group with intermediate to high scores of negative emotional symptoms at the beginning of lockdown and progressively lower scores in subsequent weeks, and a “Maladaptive” group with sustained (or increased) high scores of negative emotional symptoms throughout lockdown.

Previous work investigating mental health trajectories during lockdown identified two to five trajectories of anxiety and/or depression scores and showed that most participants exhibited sustained low scores of negative emotional symptoms over time. Conversely, a smaller portion of participants exhibited sustained high scores or worsening of negative emotional symptoms throughout lockdown, which is in accordance with our findings. (19, 21–27).

There are similarities between the mental health trajectories described in this study and those identified by Ahrens et al. (21, 37) in a longitudinal study in a German sample. These findings further support the idea that participants' negative emotional symptoms may evolve during lockdown in the described three ways. Nevertheless, in work by Ahrens et al. (21) the “recovered” group initially worsens before starting to improve mental health over time and the “delayed dysfunction” group shows significant deterioration of mental health.

Furthermore, we observed that specific individual characteristics clustered in the Maladaptive group. Therefore, it could be argued that these are risk factors for sustained high scores of stress, anxiety and depression symptoms throughout lockdown. Younger participants, participants that are not working, participants with previous mental health diagnosis, those taking psychiatric medication, smokers, and participants with a higher neuroticism score seem to be at a higher risk of maintaining high scores of stress, anxiety and depression symptoms.

In contrast, characteristics that define the Resilient group can be recognized as protective factors for stress, anxiety and depression symptoms during lockdown. This applies to higher extraversion, agreeableness and conscientiousness scores, older age, having access to a terrace or a balcony in the house, practicing exercise, and restricting consumption of COVID-19 related news to < 1 h per day.

Additionally, it is possible to identify protective and risk factors for specific negative emotional symptoms. Female participants are at higher risk for sustained high stress scores, while being male seems to be protective of stress and anxiety during lockdown. This is in accordance with previously identified gender differences showing that women have higher prevalence rates of anxiety disorders (43). It should be noted that female participants also clustered in the “recovered” group for stress scores, demonstrating greater adaptability to stress. Moreover, participants with a lower level of education, unemployed or studying cluster in the Maladaptive group for anxiety and depression symptoms.

These results are consistent with previous findings in the literature. Younger age and female sex are widely recognized as risk factors for higher stress, anxiety and depression symptoms during lockdown (2, 11, 44–48) and have been associated to “worsening” trajectories (19, 22, 26). The increased unpredictability of the future has a greater impact on young adults, whose lives are generally more prone to sudden changes. Less job security, less financial stability, and more emotional distress due to a highly affected economic and social life might explain the negative impact of COVID-19 lockdown on younger adults (49). Furthermore, this fits earlier findings supporting that younger subjects and women are more prone to mental disorders (50). The negative impact of lockdown on women's mental health might be explained by cultural differences in gender roles ingrained in Portuguese society. Traditionally, women play a key role in caring for the home, children and other dependents. In a situation where work duties add to childcare and housework roles, many women find themselves overburdened and at risk of increased stress.

Our results are in good agreement with previous findings regarding the negative impact of unemployment and the positive impact of maintaining work in emotional symptoms during COVID-19 lockdown (28, 46, 47, 51, 52). Also, financial distress, unemployment and work impairment were associated to “maladaptive” trajectories (22, 26). It has been proposed that a decrease in quality of life resulting from financial adversity increases the risk of developing adverse psychological symptoms (53). Moreover, pre-pandemic studies show that individuals with poor mental health are twice as likely to be unemployed (54). A decrease in household income is linked with an increased risk for anxiety and mood disorders (55). Fortunately, however, some measures can be implemented to prevent job loss and protect unemployed individuals. Stuckler et al. (56) showed that investments in active labor market programs focused on keeping people employed, reintegrating workers into jobs, and helping unemployed individuals cope with the negative effects of unemployment could alleviate the adverse health effects of economic downturns. Additionally, monetary support (e.g., tax deferral, wage subsidy, unemployment benefits) was suggested to mitigate unemployment's negative effects on mental health (57).

Another important finding was that students presented higher risk for persistent anxiety and depression symptoms. This piece of evidence is supported by previous findings suggesting that student status is a risk factor for developing depressive symptoms during COVID-19 lockdown (58–60) and can be explained by the distress caused by the closure of universities, postponements of exams, and remote online classes (49).

Unsurprisingly, psychiatric patients and participants taking psychiatric medication clustered in the “Maladaptive” group. These results seem to be consistent with other studies that found that patients with psychiatric disorders experienced worsening of their psychiatric symptoms during COVID-19 compulsory confinement (28, 61, 62). Moreover, having a previous mental health diagnosis has been associated with “maladaptive” trajectories (23, 24). This observation may support the hypothesis that psychiatric patients represent a vulnerable group needing added support during lockdown. Thus, increased accessibility to mental health services is crucial to mitigate the effects of compulsory confinement on psychiatric patients.

Following the present results, previous studies (including one systematic review) have demonstrated that lower education level was linked with higher anxiety and depressive symptoms during the COVID-19 pandemic (12, 47).

As expected, it is well established that smoking is associated with severe COVID-19 (63). Therefore, smokers' observed persistent negative emotional symptoms might be due to higher perceived risk and greater fear of infection.

Healthy lifestyle habits such as practicing exercise and restricting consumption of COVID-19 related news to < 1 h per day were significantly more frequent in the “Resilient” group. Recent studies also demonstrate that exercising is associated with lower stress, anxiety, and depression scores (11, 45). Pre-pandemic evidence shows that exercising can effectively alleviate and prevent anxiety and depressive symptoms (64). In addition, previous studies revealed that frequent exposure to news relating to COVID-19 is related to negative emotional symptoms (46, 65, 66). The permanent media coverage of COVID-19-related information may partly explain this finding. Additionally, the rise in misinformation and fake news can generate new fears and avoidable anxiety (67).

The existing literature also supports the link between higher neuroticism and negative Emotional symptoms (68, 69). Here, we show that a specific personality profile (high neuroticism, and low conscientiousness, extraversion, and agreeableness) cluster in the “Maladaptive” group. Interestingly, this personality profile is very similar to the one associated with depressive disorders described by Sadeq and Molinari (69). Moreover, recent works studying the COVID-19 pandemic point out the negative impact of higher neuroticism and the positive impact of higher extraversion on mental health (70, 71).

## 5 Limitations

It is plausible that several limitations may have influenced the obtained results. Our baseline measurements were performed almost 1 week after the state of emergency was declared. Therefore, additional pre-pandemic measurements could have helped us better understand the participants' changes in mental health. Stress, anxiety and depression scores obtained before the COVID-19 lockdown would allow us to compare pre-pandemic and post-pandemic values and ascertain whether or not self-reported stress, anxiety, and depression symptoms return to pre-pandemic levels. Additionally, long lasting effects of the COVID-19 pandemic might only affect participants after the end of the follow-up period. Inevitably, factors that may have a significant impact on mental health were not taken into account. This is the case of factors like loss of income despite keeping a job, use of coping strategies (e.g., meditation, reading, religious activities, gambling, and drug consumption), and housing quality. Another important limitation is that our sample is not representative of the general Portuguese adult population. It encompasses a disproportionate representation of younger and female participants and participants with a higher education level.

Moreover, since the data was collected using a series of online surveys, participants without internet access or digital knowledge are not represented. Accordingly, the generalization of the results of this study must be done carefully. Finally, we are aware that self-report psychometric instruments may lead to inaccurate estimates of symptoms (72).

## 6 Conclusions

The present study longitudinally explores trajectories of stress, anxiety and depression during COVID-19 compulsory confinement using a large sample and a robust statistical analysis. Using a LCMM we focused our analysis on discriminating differences in trajectory shape without prior assumptions of specific sample characteristics (36), which has enabled us to employ a novel approach to study the impact of COVID-19 lockdown on mental health. Identifying distinct mental health trajectories during lockdown adds important information to the hypothesis that the evolution of stress, anxiety and depression symptoms during lockdown may vary from individual to individual. Furthermore, the subsequent analyses allowed us to identify the characteristics of the individuals who present a higher risk of showing persistent negative emotional symptoms during compulsory confinement (i.e., younger age, female, lower education level, not working, studying, having a mental disorder, taking psychiatric medication, smoking). The prompt identification of those at risk of emotional suffering is essential to enable timely and effective intervention. Accordingly, a tailored approach to emotional suffering for vulnerable subjects during similar public health crises must be devised.

## Data availability statement

The raw data supporting the conclusions of this article will be made available by the authors, without undue reservation.

## Ethics statement

The studies involving humans were approved by the Ethics Committee for Research in Life and Health Sciences (CEIVCS). The studies were conducted in accordance with the local legislation and institutional requirements. The participants provided their written informed consent to participate in this study.

## Author contributions

AF: Data curation, Formal analysis, Investigation, Methodology, Writing—original draft. SF: Conceptualization, Formal analysis, Investigation, Methodology, Writing—review & editing. PM: Conceptualization, Data curation, Methodology, Writing—review & editing. MM-S: Conceptualization, Data curation, Methodology, Writing—review & editing. BC: Conceptualization, Data curation, Methodology, Writing—review & editing. CR-L: Conceptualization, Data curation, Methodology, Writing—review & editing. PC: Formal analysis, Methodology, Writing—review & editing. PM: Conceptualization, Funding acquisition, Methodology, Project administration, Resources, Supervision, Writing—review & editing. MP-P: Conceptualization, Funding acquisition, Methodology, Project administration, Resources, Supervision, Writing—review & editing.
