# Supplementary material for: Stress, anxiety, and depression trajectories during the “first wave” of the COVID-19 pandemic: what drives resilient, adaptive and maladaptive responses in the Portuguese population?
Source: Front Public Health. 2024 Feb 13;12:1333997. doi: 10.3389/fpubh.2024.1333997 (PMC10897044; doi:10.3389/fpubh.2024.1333997)
Supplement: Supplementary file 1 [file Data_Sheet_1.PDF]

ng	link function	idiag	AIC disc	Number of parameters
2	linear	FALSE	52741.97	11
		TRUE	52664.12	10
	beta	FALSE	52211.61	13
		TRUE	52133.42	12
	splines	FALSE	52057.92	16
		TRUE	52002.35	15
	5-quant-splines	FALSE	51739.17	16
		TRUE	51704.18	15
3	linear	FALSE	52647.75	14
		TRUE	52627.41	13
	beta	FALSE	52096.04	16
		TRUE	52108.64	15
	splines	FALSE	52177.01	19
		TRUE	52161.96	18
	5-quant-splines	FALSE	51882.72	19
		TRUE	51683.78	18
4	linear	FALSE	52735.12	16
		TRUE	52660.26	15
	beta	FALSE	52132.47	18
		TRUE	52161.57	17
	splines	FALSE	52070.73	21
		TRUE	52021.61	20
	5-quant-splines	FALSE	51782.84	21
		TRUE	51703.07	20

Supplementary table S1. Supplementary information regarding all estimated models for DASS-21 Stress subscale scores. ng = number of latent classes considered. idiag = logical for the variance-covariance structure of the random-effects. If idiag = FALSE, a non-structured matrix of variance-covariance is considered. If idiag = TRUE a diagonal matrix of variance-covariance is considered. AIC disc = discrete Akaike information criterion.

ng	link function	idiag	AIC disc	Number of parameters
2	linear	FALSE	38135.73	11
		TRUE	37949.74	10
	beta	FALSE	35975.62	13
		TRUE	35911.56	12
	splines	FALSE	36708.23	16
		TRUE	36655.65	15
	3-quant-splines	FALSE	35578.28	14
		TRUE	35590.10	13
3	linear	FALSE	38241.28	14
		TRUE	37950.01	13
	beta	FALSE	35973.27	16
		TRUE	35961.92	15
	splines	FALSE	36714.23	19
		TRUE	36617.24	18
	3-quant-splines	FALSE	35598.85	17
		TRUE	35633.08	16
4	linear	FALSE	38029.87	16
		TRUE	37969.64	15
	beta	FALSE	35993.25	18
		TRUE	35981.56	17
	splines	FALSE	36730.41	21
		TRUE	36632.03	20
	3-quant-splines	FALSE	35623.44	19
		TRUE	35653.34	18

Supplementary table S2. Supplementary information regarding all estimated models for DASS-21 Anxiety subscale scores. ng = number of latent classes considered. idiag = logical for the variance-covariance structure of the random-effects. If idiag = FALSE, a non-structured matrix of variance-covariance is considered. If idiag = TRUE a diagonal matrix of variance-covariance is considered. AIC disc = discrete Akaike information criterion.

ng	link function	idiag	AIC disc	Number of parameters
2	linear	FALSE	46247.79	11
		TRUE	43611.09	10
	beta	FALSE	43611.09	13
		TRUE	43576.4	12
	splines	FALSE	43744.28	16
		TRUE	43713.82	15
	5-quant-splines	FALSE	43685.17	16
		TRUE	43541.54	15
3	linear	FALSE	46253.79	14
		TRUE	46052.82	13
	beta	FALSE	43616.96	16
		TRUE	43604.16	15
	splines	FALSE	43750.28	19
		TRUE	43733.22	18
	5-quant-splines	FALSE	43569.32	19
		TRUE	43569.12	18
4	linear	FALSE	46132.17	16
		TRUE	46065.15	15
	beta	FALSE	43634.53	18
		TRUE	43634.89	17
	splines	FALSE	43767.41	21
		TRUE	43761.58	20
	5-quant-splines	FALSE	43585.46	21
		TRUE	43582.98	20

Supplementary table S3. Supplementary information regarding all estimated models for DASS-21 Depression subscale scores. ng = number of latent classes considered. idiag = logical for the variance-covariance structure of the random-effects. If idiag = FALSE, a non-structured matrix of variance-covariance is considered. If idiag = TRUE a diagonal matrix of variance-covariance is considered. AIC disc = discrete Akaike information criterion.
